# The Bark of *Picea abies* L., a Waste from Sawmill, as a Source of Valuable Compounds: Phytochemical Investigations and Isolation of a Novel Pimarane and a Stilbene Derivative

**DOI:** 10.3390/plants10102106

**Published:** 2021-10-04

**Authors:** Stefania Sut, Valeria Baldan, Marta Faggian, Irene Ferrarese, Erica Maccari, Eduardo Teobaldo, Nicola De Zordi, Paolo Bertoni, Gregorio Peron, Stefano Dall’Acqua

**Affiliations:** 1 Department of Pharmaceutical and Pharmacological Sciences, University of Padova, Via Marzolo 5, 35131 Padova, Italy; stefania.sut@unipd.it (S.S.); valeriabaldan91@gmail.com (V.B.); irene.ferrarese@unipd.it (I.F.); erica.maccari@unipd.it (E.M.); eduardo.teobaldo@studenti.unipd.it (E.T.); gregorio.peron@unive.it (G.P.); 2 Unired Srl, Via Niccolò Tommaseo 69, 35131 Padova, Italy; nutraceutica@unired.it; 3 Società Agricola Moldoi—S.A.M, SrL, Loc. Maras Moldoi 151/a, 32037 Sospirolo, Italy; ndezordi@gmail.com; 4 Holz Pichler SpA, Ega—Stenk 2, 39050 Bolzano, Italy; Paolo@holz-pichler.com

**Keywords:** *Picea abides*, bark, antioxidants, polyphenols, abietane, piceaside, LC-MS

## Abstract

In this work, the sawmill waste from *Picea abies* debarking was considered as source of valuable phytoconstituents. The extraction was performed using different ethanol/water mixtures, and characterization was obtained by LC-MS^n^. This latter revealed flavonoid glycosides, lignans, and procyanidins. Extraction with organic solvents (dichloromethane and methanol) and chromatographic separations of the obtained extracts by silica column followed by semi-preparative HPLC led to the isolation of polyphenols and terpenoids such as 21*α*-metoxy-serrat-14-en-3-one, 21*α*-hydroxy-serrat-14-en-3-one, pinoresinol, dehydroabietic acid, 15-hydroxy-dehydroabietic acid, 7-oxo-dehydroabietic acid, pimaric acid, 9*β*-pimara-7,15-dien-19-ol, 13-epi-manoyl oxide, taxifolin-3′-O-glucopyranoside, trans-astringin, and piceasides. Piceaside V and 9*β*-pimara-7-keto-19*β*-olide, two novel compounds identified for the first time in *P. abies* bark, were isolated, and their structures were elucidated using 1D and 2D NMR and MS techniques. The polyphenolic composition of the methanolic portion was also investigated using LC-MS^n^, and the piceaside content was estimated. To assess the antioxidant activity of main constituents, semi-preparative HPLC was performed on the methanolic extract, and the obtained fractions were assayed by using the DPPH test. Overall, this work shows the potential usefulness of *P. abies* bark as a source of valuable phytochemicals.

## 1. Introduction

*Picea abies* (L.) Karst (common spruce or Norway spruce) is a large evergreen coniferous species widely distributed in Northern, Central, and Eastern Europe. Spruce plays an important economical role in several countries, where its wood is widely used for the production of sawn timber and pulp [1]. During this process, large quantities of waste bark are generated and usually recycled to produce energy or burned in large furnaces [2]. However, the bark of several tree species, and in particular coniferous ones, are known to contain bioactive secondary metabolites. Our research group has previously investigated the possible use of barks or plant waste materials as a potential source of valuable compounds. The Nepalese *Abies spectabilis* was studied for its peculiar content of triterpene derivatives and as a potential source of antioxidants [3]; *Larix decidua* bark was found to be a good source of antioxidants, mainly procyanidins [4], and its dried hydro-alcoholic extract was shown to exert antimicrobial activity against respiratory-tract pathogens [5].

Norway spruce bark has been previously considered as a source of bioactive compounds, which in part are lipophilic constituents, but for the most they are made up of hydrophilic phenolics such as stilbenes, tannins, and suberin [6]. Several stilbenoids have been reported in the plant, such as piceatannol, astringin, and the more complex piceasides [7]. The main compounds that have been identified in Norway spruce bark are methyl dehydroabietate and abietic acid derivatives, as well as other terpenes, fatty acids, and waxes [8]. Such compounds present a significant interest for the pharmaceutical area. In fact, several biological activities of abietanes have been described, including antimicrobial, antifungal, antitumor, cytotoxic, antiviral, antiulcer, cardiovascular, and antioxidant, together with anti-inflammatory properties [9,10,11].

Previously published papers also report the antioxidant activity of the phenolic portion [2,12,13]. According to Li et al., more than 60 phenolic compounds have been isolated and identified from the bark of Norway spruce. Stilbenes of *P. abies* bark may occur both as aglycones and as glucosides, with astringin, isorhapontin, piceatannol, and piceid being the most representative [14,15]. Despite the reported presence of stilbenoids, resveratrol is not commonly observed in a significant amount in spruce tissue. Spruce (*Picea*) species synthetize the more complex tetrahydroxystilbenes such as astringin and isorhapontin, and resveratrol represents an intermediate in tetrahydroxystilbene glycoside biosynthesis [16].

The aim of this work was the evaluation of *P. abies* bark as a source of valuable phytoconstituents with potential usefulness in cosmetic or nutraceutical applications. Thus, extraction of dried plant material with different solvents was performed and characterization of the obtained extracts was achieved by combining LC-MS-based methods. Extraction with organic solvents (dichloromethane and methanol) and chromatographic separations of the obtained extracts were also performed to assess the presence of different classes of compounds. Finally, the antioxidant activity of the obtained extracts and fractions was assessed.

## 2. Results

### 2.1. Isolation of Phytoconstituents

The sequential extraction procedure with cyclohexane, dichloromethane, and methanol was performed by using ultrasound-assisted extraction. The procedure allowed the separation of compounds with different polarity and to investigate both lipophilic and hydrophilic portions of Norway spruce bark extract. A series of constituents were tentatively identified using the LC-MS^n^ approach and by comparison with reference compounds. Then, the isolation of main constituents was performed in order to confirm their chemical structures. Structure elucidation was achieved by 1D and 2D NMR experiments such as HSQC-DEPT, HMBC, COSY, and NOESY, as well as by high-resolution MS.

#### 2.1.1. Lipophilic Constituents from Cyclohexane and Dichloromethane Extracts

**Figure 1:**
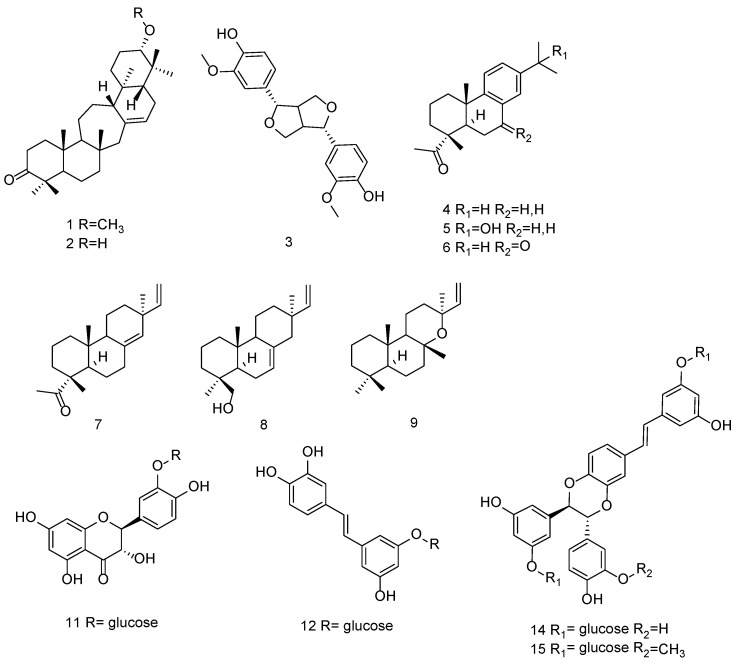
Chemical structures of compounds: 21*α*-metoxy-serrat-14-en-3-one (**1**), 21*α*-hydroxy-serrat-14-en-3-one (**2**), pinoresinol (**3**), dehydroabietic acid (**4**), 15-hydroxy dehydroabietic acid (**5**), 7-oxo dehydroabietic acid (**6**), pimaric acid (**7**), 9*β*-pimara-7,15-dien-19-ol (**8**), and 13-epimanoyl oxide (**9**), isolated from the lipophilic extracts. Taxifolin-3′-O-glucopyranoside (**11**), trans-astringin (**12**), and piceasides G and E (**14,15**) were isolated from the methanolic fraction.

Different classes of compounds were isolated from the cyclohexane and dichloromethane extracts, and their structures are reported in Figure 1. These were serratanes (**1,2**), lignan (**3**), abietanes (**4–6**), labdane (**9**), and pimaranes (**7,8,10**) derivatives. On the other hand, a flavonol (**11**), a glucoside stilbene (**12**), and three piceasides (**13–15**) were isolated from the methanolic extract.

Serratenone derivatives were obtained as light oils. APCI-MS in positive ion mode showed a protonated molecular ion [M + H]^+^ at *m/z* 455.3 for **1** and *m/z* 441.2 for **2**. The ^1^H and ^13^C NMR signals allowed the identification of known compounds [17,18]. Methoxy-serratenes appear constrained to conifers, and more specifically to Pinaceae; moreover, they are tissue-specific since they have been reported only in bark [19].

Compound **3** presented significant UV–VIS spectrum and its deprotonated molecular ion [M − H]^−^ was observed at an *m/z* of 356.9 in negative ESI-MS mode. The NMR assignments allowed it to be identified as pinoresinol, a furanoidic lignan widely distributed in conifers [20].

Compounds **4**, **5**, and **6** isolated from the dichloromethane portion were obtained as light solids, and their molecular ions [M − H]^−^ were revealed respectively at *m/z* 299.3, 315.1, and 313.0 in negative ESI-MS. These compounds exhibited similar NMR spectra characterized by the presence of three singlets and an aromatic portion. HSQC-DEPT and HMBC of compound **4** showed signals characteristic of dehydroabietic acid, such as the carboxylic group and the isopropyl group, respectively, in position 19 and 13. A deshielded singlet integration of six protons and long-range correlations for the hydroxyl group in position 15 were characteristic signals of compound **5**. Compound **6** showed deshielded aromatic signals and a keto group in position 7, confirmed by HMBC correlations between the aromatic ring and the C-6 methylene. These compounds are the dehydroabietic derivatives 15-hydroxy-dehydroabietic acid and 7-oxo-dehydroabietic acid. 

Abietanes are a family of naturally occurring diterpenoids that have been isolated from a variety of plants, included *P. abies* [18,21]. Data related to their antiproliferative activities were published [9,22], also including the antitumor effects of semisynthetic derivatives [23,24].

Pimarane derivatives were isolated from fractions of cyclohexane and dichloromethane extracts due to their lipophilic nature. Compound **8** was analyzed in positive ion mode and presented a molecular ion [M + H]^+^ at *m/z* 289.18. Negative ESI-MS showed deprotonated molecular ions [M − H]^−^ at *m/z* 301 for compound **7**, 299.1 for compound **9**, and 315.3 for compound **10**. By comparison with literature data, NMR spectra of compound **7** allowed it to be identified as pimaric acid [25]. The NMR spectra of other compounds were similar to that of **7**, suggesting that they are its derivatives. Differences are related to the geminal coupling that suggests a methylene binding with a hydroxyl group for **8** and five quaternary methyl groups and different chemical shift of the olefinic group for **9**. Literature data [26,27] also allowed the identification of 9-*β*-pimara-7,15-dien-19-ol and 13-epi-manoyl oxide as compounds **8** and **9**, respectively.

#### 2.1.2. Characterization of a Novel Pimarane Derivative from Lipophilic *P. abies* Extracts

^1^H NMR spectrum of compound **10** exhibited signals of three singlets corresponding to quaternary methyl groups at δ 1.37 (H-17), δ 1.30 (H-18), and δ 1.26 (H-20), and doublets of doublets at δ 5.67 (H-15) and δ 4.83–4.94 (H-16) ppm corresponding to sp^2^ proton signals. HSQC-DEPT spectra allowed the observation of six methylene groups at δ(H) 1.77–1.64 δ(C) 36.5, δ(H) 2.06–2.56 δ(C) 33.04, δ(H) 1.65–1.85 δ(C) 36.4, δ(H) 1.70–180 δ(C) 18.0, δ(H) 2.16 δ(C) 22.7, and δ(H) 2.24 δ(C) 24.6; 4 aliphatic C-H groups at δ 2.51 δ 46.4 (C-5), δ 4.03 δ 67.4 (C-6), δ 2.77 δ 43.6 (C-8), and δ 2.91 δ 33.7 (C-9). Further signals were present at δ 5.67 δ 146.5 (C-15), and an olefinic methylene was also observable at δ 4.83–4.94 δ 111.4 (C-16). The presence of three quaternary methyl groups and the vinyl group suggested an abietane skeleton [25,28].

The HMBC spectrum allowed the observation of diagnostic correlations between the CH_3_ 17 and the carbon resonances at δ 22.7 (C-12), δ 34.0 (C-13), δ 24.6 (C-14), and δ 146.5 (C-15). Diagnostic correlations were observed from the proton at δ 1.30 with carbons C-19, C-5, and C-3. The presence of the deshielded doublet at δ(H) 4.83 δ(C) 67.4 coupled to proton δ 2.51 (H-5) supports the presence of a lactonic ring. Long-range correlation of H-6 with C-19 (δ 180.6) confirmed this structure. The long-range correlation between H-14 and C-7 at δ 206.4 confirmed the presence of a keto group, also in position 7. From the NOESY spectrum, methyl groups 18 and 20 appeared in position *α*, instead, the 17-CH_3_ group appeared in the opposite position. The coupling constant for H-5 (J = 2.0) and the NOESY data indicated that H-5 and H-6 were from the same side and opposite to methyl groups 18 and 20. H-9 and H-8 were on the opposite side based on the measured coupling constant and NOESY correlations.

**Figure 2:**
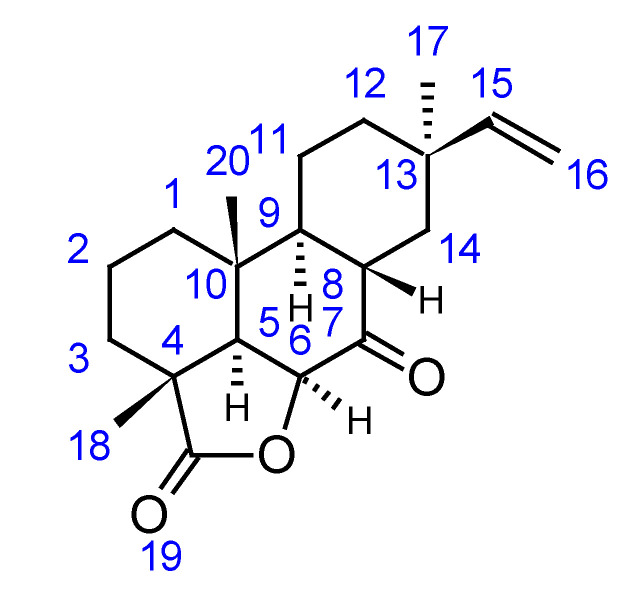
Chemical structure of compound **10**.

The NMR data indicated that the structure of this compound was characterized by a lactonic ring and a ketone group in position 7. The compound was found to be a pimarane derivative, the 9-*β*-pimara-7-keto-19-*β*-olide ((3aR,8S,10bR)-3a,8,10b-trimethyl-8-vinyldecahydro-1H-phenanthro[10,1-bc]furan-4,6(2H,3a1H)-dione), which has not been described previously. Full NMR assignments are reported in Table 1, while the chemical structure is reported in Figure 2.

#### 2.1.3. Hydrophilic Constituents from Methanolic Extract

Finally, from the methanolic extract, more hydrophilic compounds (flavonolic and stilbenoid derivatives) were isolated. Compound **11** proved to be taxifolin-3′O-*β*-D-glucopyranoside, while compound **12** was a stilbenoid glucoside, the trans-astringin. These compounds are known phytocomponents of spruce bark [1,2,29].

Molecular ions of compounds **13** and **14** were found at *m/z* 809.5 and 809.6, respectively, in negative ESI-MS. Compound **15** was analyzed in positive mode and showed an [M + Na]^+^ adduct of *m/z* 847.6.

They presented similar NMR spectra, with a complex portion of aromatic signals. According to Li et al., compounds **14** and **15** were identified as a mixture of piceaside G/H and of piceaside E/F.

#### 2.1.4. Characterization of the Novel Stilbenoid Piceaside V from *P. abies* Methanol Extract

The HR-ESI-MS of compound **13** showed the pseudomolecular ion [M − H]^−^ at *m/z* 809.7439, consistent with molecular formula C_40_H_41_O_18_ (calculated *m/z* = 809.7433). The UV–Vis spectrum showed λ_max_ at 328, 310, and 285. ^1^H-NMR spectrum showed several singlets integrating for one proton at δ 6.34, 6.42, 6.30, 6.54, 6.49, and 6.76, corresponding to two 1,3,5-trisubstituted aromatic rings. Further signals are the doublets at δ 6.74 and 6.84 and a doublet of doublets at δ 6.68, suggesting the presence of a 1,3,4-trisubstituited ring.

From the HSQC-DEPT spectra we observed 12 C-H groups, 2 olefinic C-H groups, and 2 deshielded C-H groups with δ(H) 4.40, 5.41 and δ(C) 58.1 and 93.8. We also observed two anomeric C-H groups suggesting the presence of sugar moieties, and two signals corresponding to *trans* protons of a double bond at δ 7.05 (C-7) and δ 6.97 (C-8). Diagnostic correlations could be observed in HMBC spectra between δ 6.68 (H-6′) and δ 93.8 (C-7′) and 145.5 (C-4″), 112.5 (C-2″), and δ 114.8 (C-5′). H-2′ is correlated with carbons at δ 144.5 (C-3′), 93.8 (C-7′), and 131.6 (C-1′). Signals at δ(H) 4.40 with δ(C-8′) 58.1 and δ(H) at 5.41 with δ(C-7′) 93.8. These data suggested the presence of a furanic group, also confirmed by the correlations between H-8′ with C-5 and H-7′ with C-4, assigning the link of furan group with an aromatic ring. Correlations between anomeric protons at δ 4.80 (H-1″) and δ 4.78 (H-1‴) with carbons C-11 (δ 159.5) and C-4′ (δ145.5) allowed the definition of the glycosylation positions.

**Figure 3:**
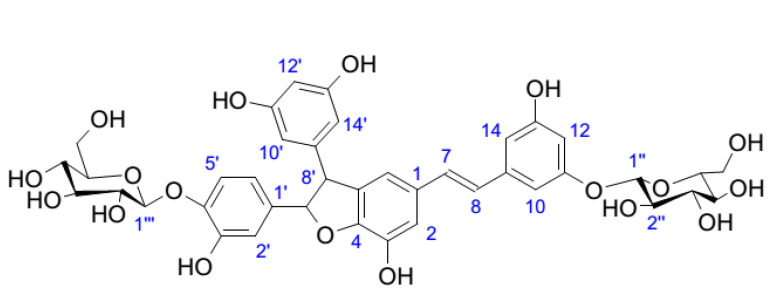
Chemical structure of compound **13**, piceaside V.

Despite the HPLC separation, compound **13** appeared to be a mixture of different diastereoisomers that are not separable from each other as reported by Li et al. [7]. On the basis of these literature data, this compound belongs to the class of piceasides, and its structure is similar to piceaside A, except for the position of the sugar moiety on C-4′ and not on C-11′. Sugar portions were determined using the hydrolysis protocol previously reported in [30], and they were assigned to *β*-D-glucopyranosides. Considering all these data, compound **13** was named piceaside V. Full NMR assignments are reported in Table 2, while the chemical structure is reported in Figure 3.

### 2.2. LC-DAD-MS^n^ Analysis of Methanol Extract

Methanol extract was analyzed by LC-DAD-MS^n^. Compound identification was performed by comparing their fragmentation patterns with isolated reference compounds or with published literature [7,15,31]. Flavonoids such as taxifolin and its derivatives, stilbenes, and piceasides were the main constituents. In Table 3 are reported all the identified compounds, along with their chromatographic retention times and ions produced by MS^n^ fragmentation.

**Figure 4:**
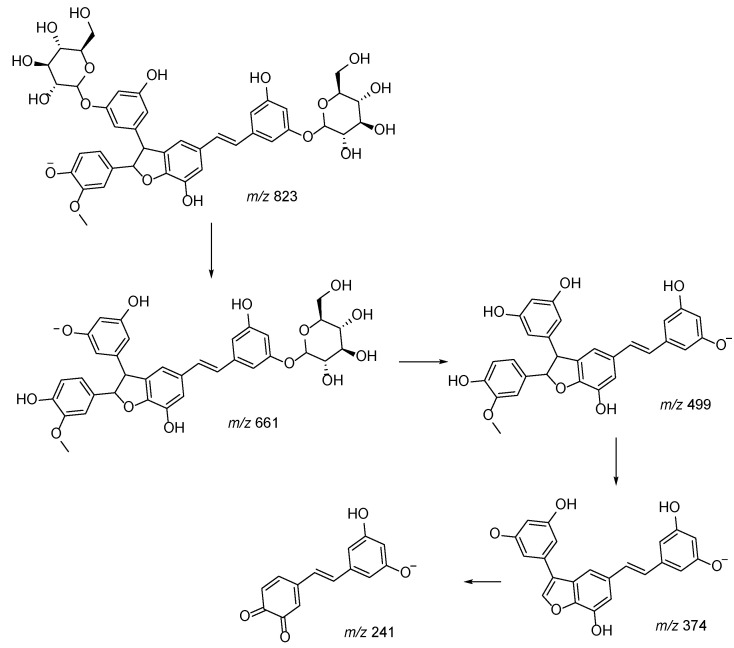
Proposed fragmentation pattern of piceaside with furanic group.

We observed the presence of different piceaside isomers, and the MS^n^ approach allowed clarification of the fragmentation pathways by comparison with data obtained by NMR analysis of the isolated compounds. Two different behaviors can be observed for compounds presenting molecular ion [M − H]^−^ at *m/z* 809. MS^n^ fragmentation of derivatives with a dioxanic group forms ions at *m/z* 647, 405, and 243. This is probably due to the presence of two C-O bonds that facilitate the break with formation of the product ion at *m/z* 243, corresponding to the *trans*-astringin nucleus, and the loss of the sugar moiety leading to the formation of piceatannol. On the other hand, the species presenting molecular ion [M − H]^−^ at *m/z* 809 generating fragments in MS^n^ at *m/z* 647, 485, 375, and 241 were characterized by the presence of the furanic cycle, as reported in Figure 4 (R = H). In this case, the loss of sugar moieties was favored over breaking of the aglycone portion.

**Figure 5:**
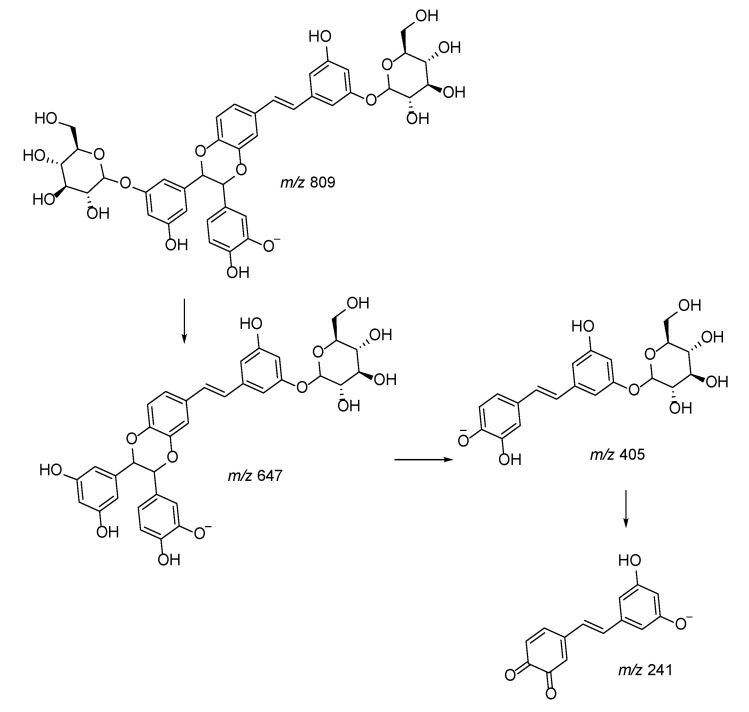
Proposed fragmentation pattern of piceaside with dioxanic group.

In the same way, thanks to their different fragmentation patterns, we tentatively differentiated methoxy piceasides C/D and E/F (*m/z* 823), reported in Figure 5 as R = CH_3_.

The amount of piceasides and flavonoids in methanolic bark extract were estimated by the HPLC-DAD method. Due to the lack of reference compounds, resveratrol was used as an external standard for the quantification of piceasides, and the peaks were determined on the basis of the UV spectra compared with resveratrol signal. Of note, if authentic standards are not available, similar compounds may be used for external calibration to estimate the amounts of other compounds [15].

Flavonoid quantification was carried out using the method previously reported [5], and rutin was used as the reference compound. The calibration curves were established by analyzing five methanolic rutin solutions (8–80 μg/mL), and the DAD chromatogram was monitored at λ = 350, 330, and 280 nm. Results are reported in Table 4.

### 2.3. Antioxidant Properties of Methanolic Extract

The free radical scavenging properties of *P. abies* methanolic extract were evaluated using the DDPH assay. Free radicals are highly reactive species that have at least one unpaired electron. Reactive oxygen species (ROS) react with free radicals to become radicals themselves. DPPH is a stable free radical that can accept an electron, becoming a stable molecule [32]. The decrease of DPPH absorbance at 517 nm indicates an increased percentage of free radical scavenging activity. For this assay, ascorbic acid was used as the reference substance.

The total methanolic extract presented a proton-donating ability and showed a good scavenging capacity, with IC_50_ = 12.10 ± 1.41 µg/mL. Comparison with ascorbic acid response (IC_50_ = 3.34 ± 0.03 µg/mL) indicates that *P. abies* methanol extract could serve as free radical inhibitor or scavenger, acting possibly as a primary antioxidant. In order to investigate which compounds are mainly responsible for this activity, a semi-preparative HPLC was performed, and the total extract was divided into fractions with the same volume. The 20 fractions obtained were then subjected to DPPH assay.

The elution solvent used for the fractionation was used as a blank solution.

**Figure 6:**
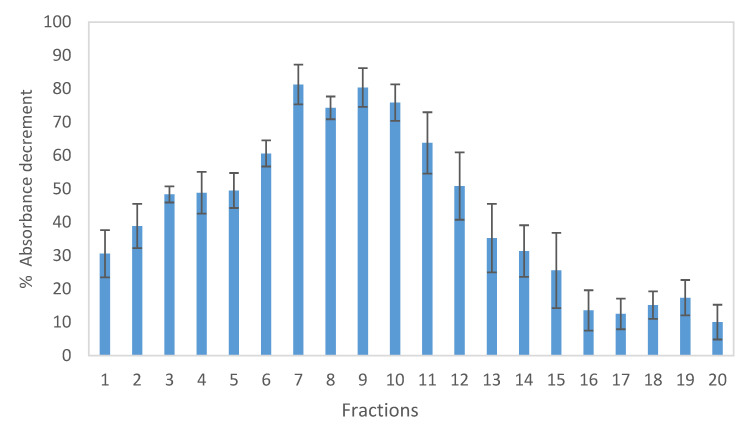
Absorbance decrement (%) after DPPH reaction of methanolic extract fractions.

After the incubation of DPPH with each fraction, the absorbance was read at 517 nm, and the results were expressed as percentage of decrement in absorbance compared with the eluent (Figure 6). Fractions 7, 8, 9, and 10 showed the highest response: 81.3, 74.3, 80.4, and 75.8% of absorbance decrement, respectively.

### 2.4. LC-DAD-MS^n^ Characterization of Fractions Isolated from Methanol Extract Showing Higher Scavenging Activity against DPPH

Fractions 7, 8, 9, and 10 obtained by the semi-preparative HPLC of the *P. abies* methanol extract were analyzed by HPLC-MS to identify the main constituents, which were then quantified using HPLC-DAD, as previously reported for the whole extract. The chemical composition of each fraction was then associated with the corresponding antioxidant activity observed from the DPPH assay. This approach is a simple method that allows the identification of the compounds that react with free radicals, and thus, which compound(s) are involved in the antioxidant activity. In fact, the decrement in absorbance is correlated with the scavenging activity of antioxidants [33].

The HPLC-MS analysis revealed astringin, taxifolin-7-O-glucoside, piceaside A/B, piceaside G/H, and mono-methoxy-piceasides as the main components. The data are in agreement with the previous characterization of the total extract. Compound amounts were estimated by DAD data using resveratrol as the reference compound. Although the standards for astringin and taxifolin are available, the quantification was achieved using resveratrol to easily compare the data. The amounts are reported in Table 5.

The four considered fractions showed different amounts of active compounds, with a total concentration ranging from 9.0 to 37.5 μg/mL. Comparing these data with the percentage of scavenging effects, fractions 9 and 10 were found to have the most antioxidant activity, in fact they presented a high DPPH scavenging effect with a low amount of phenolic compounds.

The antioxidant activity of taxifolin and astringin derivatives is widely reported in literature [34,35,36]. Piceasides were found to be the most significant antioxidant compounds present in the methanolic extract. The scavenging capacity of piceasides may be related to their piceatannol moiety, which is a known antioxidant compound [37]. Further investigations on the isolated compounds are necessary to verify the contribution of each one in the antioxidant properties of the extract.

## 3. Discussion

The present study aimed to fully characterize the phytochemical components of Norway spruce bark waste, and results indicate its possible use as source of phytoconstituents. Other groups have previously considered this bark as valuable source of phytoconstituents [38,39,40], but our work and results give new approaches in the exploitation of this waste. The approach based on sequential extractions allowed the characterization of several classes of compounds and led to a more complete knowledge of the chemical composition of Norway spruce bark extract. Furthermore, our approach offers the opportunity to plan also the extraction of lipophilic compounds that, up to now, have been poorly considered for cosmetic and nutraceutical applications. We achieved the isolation of different *P. abies* bark constituents such as stilbene and abietane derivatives that were previously reported in the literature [9,41]. These compounds can be considered as valuable substances, in fact their biological activities have been widely reported by other authors [9,11,14,42]. A novel finding of this work is the isolation of two new compounds, and this result indicates that further studies are needed to deepen the knowledge regarding Norway spruce bark composition. Furthermore, it suggests that storage of the bark can also contribute to chemical modification of some of the constituents, as previously indicated [40], since the highly oxygenated new terpenoids may be formed during bark storage. This paper reports the isolation of 15 main constituents, namely 21*α*-metoxy-serrat-14-en-3-one (**1**), 21*α*-hydroxy-serrat-14-en-3-one (**2**), pinoresinol (**3**), dehydroabietic acid (**4**), 15-hydroxy dehydroabietic acid (**5**), 7-oxo dehydroabietic acid (**6**), pimaric acid (**7**), 9*β*-pimara-7,15-dien-19-ol (**8**), and 13-epimanoyl oxide (**9**) from the lipophilic extracts, and taxifolin-3′-O-glucopyranoside (**11**), trans-astringin (**12**), and piceasides G and E (**14,15**) from the methanolic fraction. The compounds 9*β*-pimara-7-keto-19*β*-olide (**10**) and piceaside V (**13**) were isolated from the lipophilic and methanol fractions, respectively, and are here described in *P. abies* bark for the first time. Compared to the other diterpenes characterized in *P. abies* bark, compound **10** shows a higher degree of oxidation. Considering that the bark removed from the felled trees was stored for 3 weeks before extraction, we cannot exclude that this novel derivative could have formed from the oxidation of other diterpenoids among those already identified in *P. abies* bark. Further studies aimed at studying the effect of storage conditions on the chemical composition of this natural material are needed.

Overall, the analytical results obtained in this study indicate that timber industry wastes such as *Picea* bark can represent a significant source of potentially bioactive chemical constituents, and a reliable extraction of such compounds may be planned from these materials.

The methanolic extract presents more than 3% of piceaside derivatives and the LC-DAD-MS^n^ analysis revealed the presence of different phenolics. All these compounds can be considered as a significant phytocomplex with potential usefulness in cosmetic applications or as an antioxidant.

Further results indicate the scavenging capacity of both total extract and fractions toward DPPH reagent. Piceasides have been identified as one of the compounds responsible for free radical scavenging capacity, together with astringin and taxifolin derivatives. 

Although more studies are needed, the overall data indicate *P. abies* bark is a source of valuable phytoconstituents for nutraceuticals and cosmetic applications.

## 4. Materials and Methods 

### 4.1. Plant Material

*P. abies* bark was obtained as residue of the timber industry kindly provided by Holz Pichler (Belluno, Italy). A voucher specimen (code: AB001) has been deposited at the Department of Pharmaceutical and Pharmacological sciences of the University of Padua.

### 4.2. Extraction and Fractionation of Dried Plant Material

Finely dried powdered bark (400 g) was extracted in an ultrasound bath with reflux apparatus using sequential solvents at increasing polarity: cyclohexane, dichloromethane, and methanol. Extractions were performed under stirring for 1 h at room temperature and with a dried plant material:solvent ratio of 1:10. The extracts were then filtered, and solvents were removed to dryness using a rotary evaporator. The yields of extraction were 1.63, 2.52, and 3.86% *w*/*w*, respectively.

Each extract was loaded in a flash chromatography column and eluted in isocratic mode using the more appropriate solvent (or mixtures) for each ones: cyclohexane for cyclohexane extract, cyclohexane:acetone 90:10 for dichloromethane extract, and chloroform:methanol 70:30 for the methanolic one. Chromatograms were recorded at λ = 254, 280, and 350 nm. 

From the cyclohexane extract (6.53 g), we obtained 33 fractions that were spotted on a TLC (Merck) and eluted using a cyclohexane:ethyl acetate 6:1 mixture as solvent, in order to evaluate their behavior and bring together similar fractions. A UV lamp at λ = 254 and 365 nm was used to visualize the TLC spots. In this way, we obtained 20 fractions, namely from A to V. Further purification steps on TLC and semi-preparative HPLC were performed. From the fractions D, G, and U, we obtained compounds **2**, **7**, and **9** with a weight of 9.3, 5.5, and 3.3 mg, respectively.

Dichloromethane extract (10.08 g) was fractionated into 13 fractions (1–13) and the methanolic extract in nine fractions (A–I). All these fractions were subjected to several purification steps on TLC using 70:30 toluene:acetone and 10:5:1 chloroform:methanol:water mixtures as eluents, respectively. Fractions 5 and 8 from dichloromethane extract were found to be pure enough, instead an LC semi-preparative analysis was performed to purify fractions 6 and 10 using the method reported below. The isolated compounds were **1**, **3**, **4**, **5**, **6**, **8**, and **10**. The weights of the pure compounds were 6.7, 2.8, 20.4, 5.4, 2.5, 8.0, and 0.7 mg, respectively.

From fraction E of the methanolic extract, we obtained the compound **11**. From fraction F, we isolated four compounds using the semi-preparative LC method, namely compounds **12**, **13**, **14**, and **15**.

Methanolic extract was also fractionated into 20 fractions by LC semi-preparative method using the elution gradient for hydrophilic samples (reported below). These fractions were then analyzed for their antioxidant properties using the DPPH test.

### 4.3. Flash Chromatography 

Fractionation of extracts was performed on Flash Purification System IntelliFlash Varian 971-FP equipped with quaternary pump, UV detector, and fraction collector. Chromatograms were monitored at λ = 254, 280, and 350 nm. The elution was set on isocratic mode, using cyclohexane, 90:10 cyclohexane:acetone mixture, and 70:30 chloroform:methanol mixture as eluents for cyclohexane, dichloromethane, and methanolic extracts, respectively. SuperFlash Analogix SF 25-120 g column cartridges were used.

Methanolic fractions were collected, setting 15 mL as the collection volume.

### 4.4. Preparative Liquid Chromatography 

The LC preparative system consisted of a Varian 920 chromatograph equipped with a quaternary pump. Chromatographic separation was achieved on a LiChroCART 250-10 column. For lipophilic fractions, the mobile phase was delivered at a flow rate of 5 mL/min. The chromatographic run was performed with a binary, linear A/B gradient (solvent A: 1% formic acid in water; solvent B: acetonitrile). The elution program was as follows: 0 min, 10% B; 20 min, 90% B; 32 min, 90% B; 33 min, 10% B, and isocratic up to 40 min. The injection volume was 100 μL, and the chromatograms were monitored at 254 nm. 

The hydrophilic fractions were analyzed using a different elution gradient, i.e., a linear A/B gradient of 1% formic acid in water and methanol. Elution gradient was as follows: 0 min, 5% B; 20 min, 85% B; 25 min, 85% B; 26 min, 5% B, and isocratic up to 32 min. The flow rate was 4.6 mL/min. The injection volume was 100 μL, and the chromatograms were monitored at 280 nm.

Each peak was collected, and the obtained fractions were evaporated to dryness under vacuum on a rotary evaporator at 40 °C. The dry residues were re-dissolved in deuterated solvent for ^1^H-NMR analysis.

NMR spectra were obtained on a Bruker Avance 400 spectrometer, using standard pulse sequences. Samples were prepared by dissolving the dry extract in deuterated solvent.

### 4.5. Direct MS Infusion and NMR Spectroscopy

To confirm the structure and the mass of each isolated compound, the samples were injected on a Varian MS-500 ion trap MS. Spectra were recorded in negative or positive ion mode in the 50–2000 Da range, using both APCI and ESI ion sources.

NMR spectra were obtained on a Bruker AVANCE 3 instrument operating at 400 MHz for ^1^H. Spectra were acquired at 25 °C using standard Bruker pulse programs. For each sample, P1 and D1 were measured before spectra acquisition. Two-dimensional NMR experiments, namely Heteronuclear Single Quantum Coherence (HSQC) with Distortionless enhancement by polarization transfer editing, Heteronuclear Multi Bond Correlation (HMBC), Correlation Spectroscopy (COSY), Nuclear Overhauser Effect Spectroscopy (NOESY), and Total Correlation Spectroscopy (TOCSY), were used to assign the structure of compounds.

### 4.6. High-Performance Liquid Chromatography–Diode Array Detector–Mass Spectrometry (HPLC-DAD-MS^n^)

Quali-quantitative analysis of flavonoids and piceasides was performed using an HPLC-DAD-MS^n^ system. Measurements were obtained with an Agilent 1260 chromatograph equipped with a 1260 diode array and a Varian MS-500 ion trap (Santa Clara, CA, USA) as detectors. An Eclipse XDB C-8 2.1 × 150 mm 3.5 μm (Agilent) column was used as the stationary phase and acetonitrile (A) and 0.1% formic acid in water (B) were used as mobile phases. The elution gradient was set as follows: linear gradient from 90% B to 40% B, 0–45 min; linear gradient from 40% B to 0% B, 45–51 min; isocratic gradient 0% B, 51–55 min; linear gradient from 0% B to 90% B, 55–56 min, and isocratic gradient until 60 min. The flow rate was 0.4 mL/min, and the injection volume was 10 μL. At the end of the column, a T connector was used to split the flow from the column to DAD and MS. DAD chromatograms were detected at different wavelengths (350 and 280 nm) according to the absorption maxima of analyzed compounds, and the UV–VIS spectra were acquired in the λ range of 200–650 nm. MS spectra were recorded in negative ion mode in the 50–2000 Da range, using an ESI ion source. The turbo data depending scanning (TDDS) function allowed the fragmentation of the main ionic species. Identification of compounds was based on the fragmentation spectra, as well as the comparison of the fragmentation pattern with the literature and injection of reference compounds, when available. All flavonoids and piceasides were quantified using the external standard method. Quantification was based on peak area (DAD). Calibration curves of the standards were prepared by diluting stock standard solutions in methanol to yield final concentrations of 8–80 µg/mL (rutin) and 7.2–72 µg/mL (resveratrol). Linear regressions were as follows: for rutin, y = 15.101x + 6.3224 (R^2^ = 0.999); for resveratrol, y = 0.007x − 1.046 (R^2^ = 0.999).

### 4.7. DPPH Assay

The free radical scavenging ability of the total methanolic extract and its fractions against DPPH (1,1-diphenyl-2 picrylhydrazyl) free radicals were evaluated using the methodology previously described [5]. The methanolic DPPH solution (50 µg/mL) was prepared daily, and 1 mL of 2-, 5-, or 10-fold diluted extract in methanol was mixed with 3 mL of DPPH solution. After the incubation (30 min in the dark), the adsorbance was acquired at λ = 517 nm. A standard curve of ascorbic acid was prepared by determining the decrease in absorbance of the DPPH radical solution compared to the blank solution. DPPH assay on methanolic fractions was performed using the elution solvent as a blank, and the decrement of absorbance of the DPPH fraction was compared to that of the blank. The data were then expressed as percentage of absorbance decrement using the formula:

(A_blank_ − A_sample_)/A_blank_ × 100.

## 5. Conclusions

In the present work, residue from the debarking process of a sawmill composed of *P. abies* bark was used as starting material for the extraction of valuable phytoconstituents. Extraction with lipophilic solvent allowed the isolation and identification of terpenoids, and from these extracts two new highly oxygenated derivatives were obtained. Previous studies indicated stilbenoids as abundant constituents of the *P. abies* bark, but their levels can be strongly influenced by conservation and storage. The material considered in the present work contained abietanes, piceasides, and flavonoids. Furthermore, two novel compounds belonging to the families of stilbenes and diterpenes were isolated, and their structure was elucidated by using both NMR and MS. Overall, the data reported in this article indicate that *P. abies* bark recovered from sawmill by-products is a valuable source of potentially bioactive compounds with antioxidant properties, as demonstrated by the DPPH assay. Future studies will be focused on the industrialization process and will also consider the effect of storage time and environmental conditions on the chemical composition of *P. abies* bark, especially to assess potential transformations of its constituents due to oxidation processes. Finally, further research efforts will address the application of whole *P. abies* bark extracts and/or isolated compounds in cosmetic and nutraceutical fields.

## Figures and Tables

**Table 1:** ^1^H and ^13^C NMR data of compound **10**.

Position	^1^H δ (Mult.)	^13^C δ (C Type)
**1**	1.64–1.77 (2H)	36.5 (CH_2_)
**2**	2.06–2.36 (2H)	33.08 (CH_2_)
**3**	1.65–1.85 (2H)	36.4 (CH_2_)
**4**	-	36.7
**5**	2.51 (d, 2Hz, 1H)	46.4 (CH)
**6**	4.03 (d, 2Hz, 1H)	67.4 (CH)
**7**	-	206.4
**8**	2.71 (m, 1H)	43.6 (CH)
**9**	2.91 (dd, J = 10. 3; 9; 1H)	33.7 (CH)
**10**	-	38.9
**11**	1.70–1.80 (m, 2H)	18.0 (CH_2_)
**12**	2.16 (m, 2H)	22.7 (CH_2_)
**13**	-	34.0
**14**	2.24 (2H)	24.6 (CH_2_)
**15**	5.67 (d, J = 15.5; 1H)	146.5 (CH)
**16**	4.83–4.94 (dd, J = 15.5; 3.3; 2H)	111.4 (CH_2_)
**17**	1.37 (s, 3H)	16.2 (CH_3_)
**18**	1.30 (s, 3H)	23.9 (CH_3_)
**19**	-	180.6
**20**	1.26 (s, 3H)	26.22 (CH_3_)

Assignments based on DEPT, COSY, HSQC, HMBC, and NOESY experiments.

**Table 2:** ^1^H and ^13^C NMR data of compound **13**.

Position	^1^H δ (mult.)	^13^C δ (C type)
**1**	-	131.3
**2**	6.77 (d, J = 1.0; 1H)	115.2 (CH)
**3**	-	149.2
**4**	-	145.8
**5**	-	132.3
**6**	6.70 (d, J = 1.0; 1H)	114.2 (CH)
**7**	7.03 (d, J = 15.5, 1H)	128.3 (CH)
**8**	6.81 (d, J = 15.5 1H)	125.1 (CH)
**9**	-	-
**10**	6.76 (d, J = 1.1; 1H)	105.1 (CH)
**11**	-	159.5
**12**	6.49 (d, J = 1.1; 1H)	105.5 (CH)
**13**	-	159.5
**14**	6.54 (d, J = 1.1; 1H)	105.3 (CH)
**1′**	-	131.6
**2′**	6.80 (d, J = 1.2; 1H)	112.5 (CH)
**3′**	-	144.5
**4′**	-	145.5
**5′**	6.74 (d, J = 7.5; 1H)	114.8 (CH)
**6′**	6.68 (dd, J = 7.5, 1.1; 1H)	116.7 (CH)
**7′**	5.41 (d, 1H)	93.8 (CH)
**8′**	4.40 (d, 1H)	58.1 (CH)
**9′**	-	143.8
**10′**	6.42 (d, J = 1.0; 1H)	109.6 (CH)
**11′**	-	158.7
**12′**	6.30 (d, J = 1.0; 1H)	104.1 (CH)
**13′**	-	161.5
**14′**	6.34 (d, J = 1.0; 1H)	107.9 (CH)
**1″**	4.80 (1H; J = 7.0)	101.2
**2″**	3.47 (1H)	73.4
**3″**	3.39 (1H)	76.7
**4″**	3.32 (1H)	70.2
**5″**	3.65 (1H)	76.5
**6″**	3.95–3.71 (2H)	60.4
**1‴**	4.78 (1H; J = 7.2)	101.2
**2‴**	3.48 (1H)	73.5
**3‴**	3.39 (1H)	75.3
**4‴**	3.33 (1H)	69.4
**5‴**	3.65 (1H)	75.3
**6‴**	3.85–3.71 (2H)	60.8

Assignments based on DEPT, COSY, HSQC, HMBC, and NOESY experiments.

**Table 3:** Hydrophilic constituents of *P. abies* methanolic bark extract identified by HPLC-MS^n^.

Compound	Retention Time (min)	[M − H]^−^ *m/z*	ESI-MS^n^ *m/z*
Hydroxy-piceaside derivative	1.42	665	485-443-305-243
Benzoic acid derivative	1.44	313	151-282
Caffeoyl-hexoside	1.48	341	203-179-131
Quinic acid *	1.54	191	127-111
Caffeic acid derivative	1.60	377	341-179
Procyanidin trimer B	1.93	865	695-577-407
Protocatechuic acid-hexoside	2.20	315	153-109
Ferulic acid *	4.17	193	173-145
(epi)-Catechin *	4.32	289	245-203
Hydroxy-piceaside derivative	4.40	665	485-443
Isorhamnetin *	5.53	315	299
Taxifolin-7-O-glucoside *	5.72	465	447-303-285
Luteolin-7-O-rhamnoside *	5.80	431	285-241
Hydroxy-piceaside derivative	6.12	665	485-443-305
Trans-astringin *	6.71	405	243
Hydroxy-piceaside derivative	7.20	665	503-445-297
Ellagic acid hexoside	7.31	463	301
Piceaside A/B	7.57	809	647-485-375
Hydroxy-piceaside derivative	8.22	665	503-445-297
Piceaside A/B	9.16	809	647-485-375-229
Isorhapontigenin	10.14	257	241-213
Piceatannol	10.73	243	225-201
Hydroxy-piceaside derivative	11.13	665	503-445-243
Piceaside A/B	11.20	809	647-485-375
Piceaside A/B	11.61	809	647-485-375-318
Piceaside G/H	12.42	809	646-405
Piceaside C/D	12.56	823	661-499
Piceaside C/D	13.73	823	661-499
Piceaside C/D	14.22	823	661-499
Piceaside G/H	14.95	809	646-405-243
Piceaside C/D	15.27	823	661-499-257
Taxifolin *	16.41	303	285-241-213
Isorhamnetin-pentoside	16.66	447	315-300
Piceaside E/F	16.70	823	661-499-241
7-hydroxy-matairesinol *	17.64	373	355-311-296
Piceaside G/H	18.39	809	646-405
Piceaside E/F	18.57	823	661-499-241
Piceaside G/H	19.13	809	646-405-243
Piceatannol derivative	19.17	647	485-243
Methoxy-piceatannol hexoside	20.25	661	499-241
Piceaside E/F	21.47	823	661-499-241
Methoxy-piceatannol	23.43	499	467-389-241
Quercetin *	52.13	301	179-151

* Confirmed by comparison with reference standard.

**Table 4:** Quantity of flavonoids and piceasides in bark of Norway spruce methanolic extract.

Chemical Class	% *w*/*w*
Flavonoids, expressed as rutin	0.32 ± 0.01
Piceasides, expressed as resveratrol	3.56 ± 0.05

**Table 5:** Amounts of phenolic compounds in the main fractions obtained from methanolic extract.

No. of Fraction	Identified Compounds	μg/mL	Total μg/mL
7	Astringin Piceaside A/B Taxifolin-7-O-glucoside Piceaside G/H	2.32 ± 0.05 14.68 ± 0.05 9.65 ± 0.07 10.82 ± 0.02	37.47 ± 0.20
8	Astringin Piceaside A/B Taxifolin-7-O- glucoside Piceaside G/H	1.97 ± 0.01 13.11 ± 0.12 3.77 ± 0.02 6.82 ± 0.05	25.67 ± 0.18
9	Piceaside A/B Piceaside G/H	6.74 ± 0.10 2.16 ± 0.01	8.90 ± 0.07
10	Methoxy-piceatannol	-	9.0 ± 0.02

## Data Availability

All data generated or analyzed during this study are included in this published article.
